# Supplementary figures and images for: Metagenomic chromosome conformation capture (meta3C) unveils the diversity of chromosome organization in microorganisms
Source: eLife. 2014 Dec 17;3:e03318. doi: 10.7554/eLife.03318 (PMC4381813; doi:10.7554/eLife.03318)

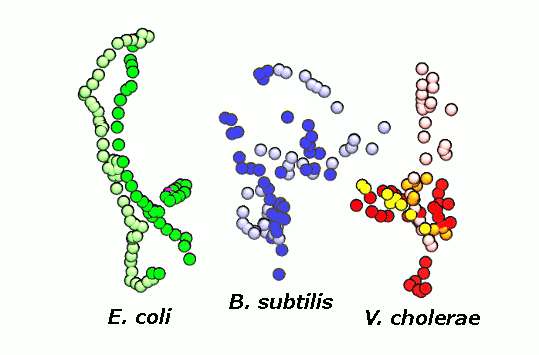

Supplement: Supplementary file 1 [file elife-03318-media1.gif]

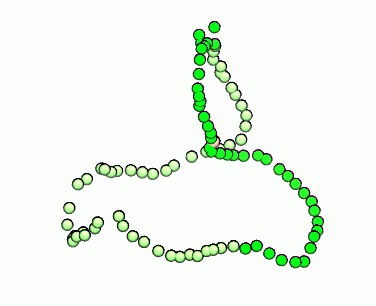

Supplement: Supplementary file 2 [file elife-03318-media2.gif]

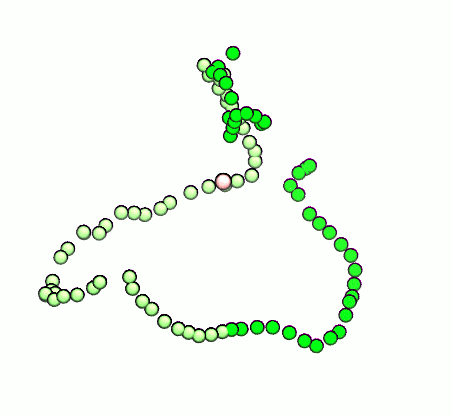

Supplement: Supplementary file 3 [file elife-03318-media3.gif]

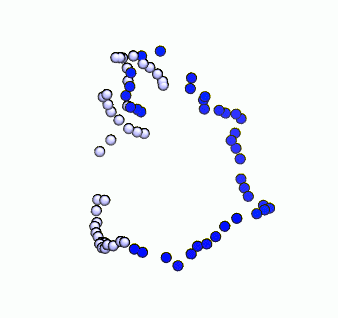

Supplement: Supplementary file 4 [file elife-03318-media4.gif]

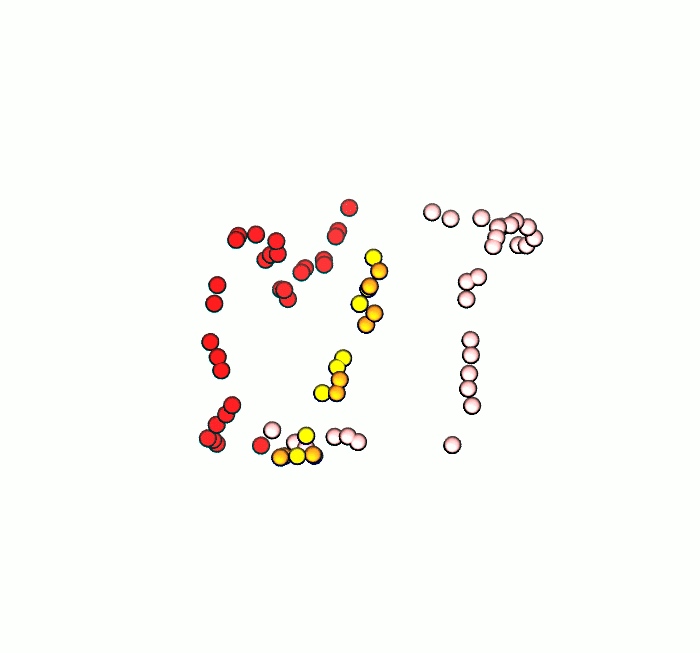

Supplement: Supplementary file 5 [file elife-03318-media5.gif]

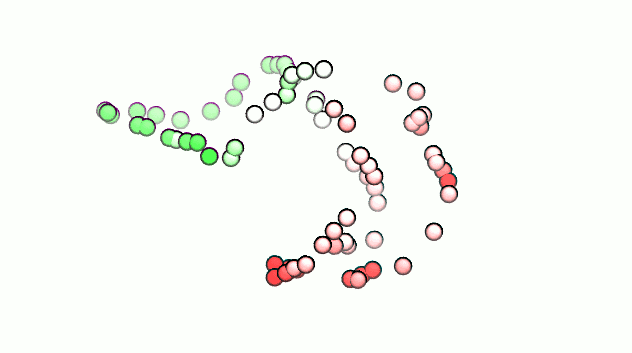

Supplement: Supplementary file 6 [file elife-03318-media6.gif]

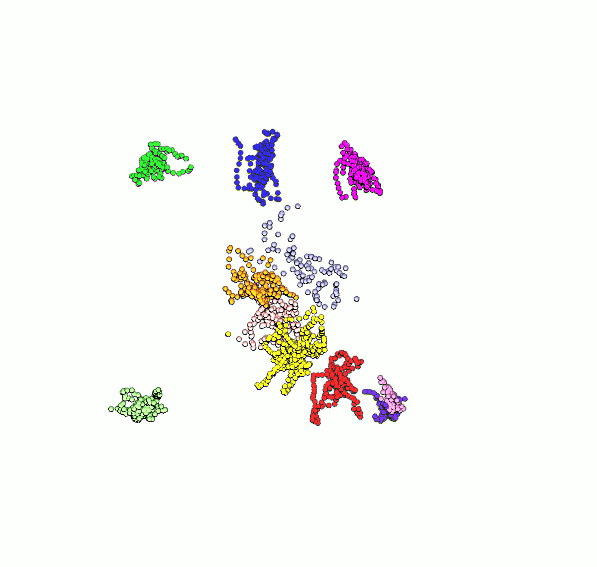

Supplement: Supplementary file 7 [file elife-03318-media7.gif]

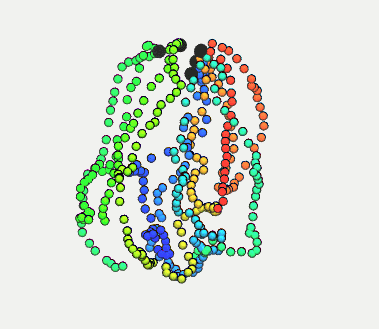

Supplement: Supplementary file 8 [file elife-03318-media8.gif]

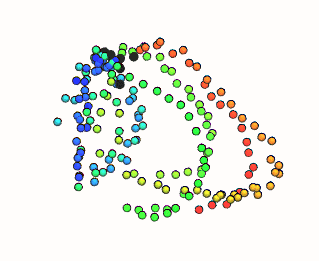

Supplement: Supplementary file 9 [file elife-03318-media9.gif]

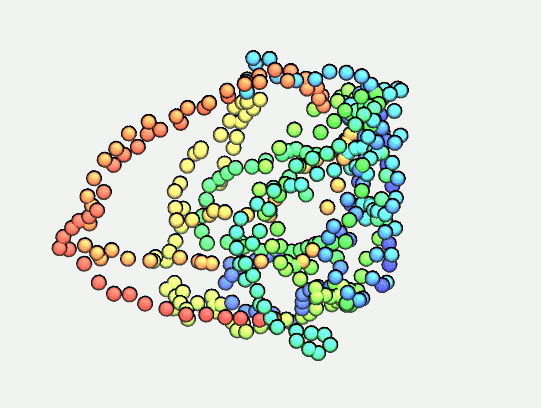

Supplement: Supplementary file 10 [file elife-03318-media10.gif]

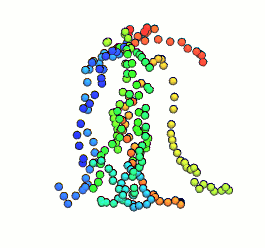

Supplement: Supplementary file 11 [file elife-03318-media11.gif]

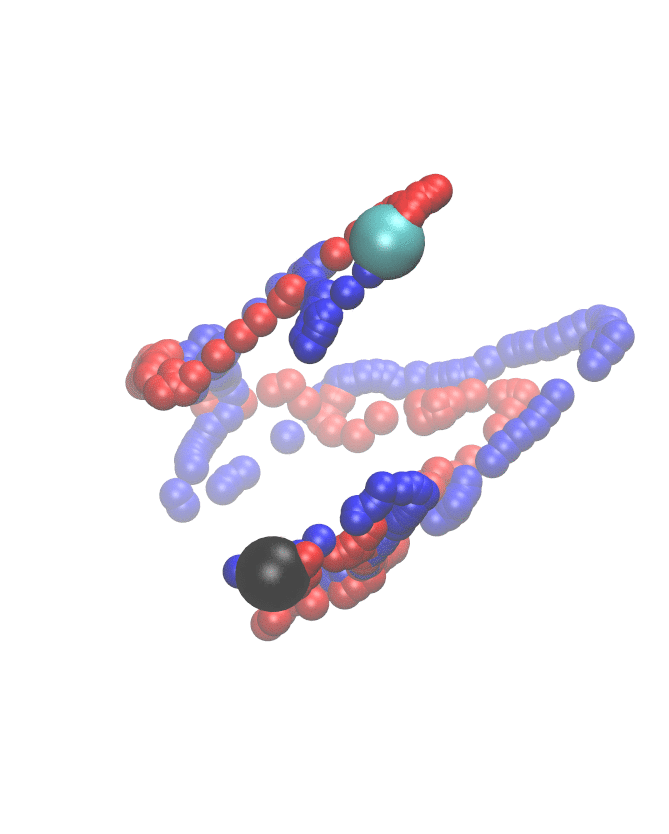

Supplement: Supplementary file 12 [file elife-03318-media12.gif]
